# Supplementary figures and images for: Short- versus long-term dual antiplatelet therapy after second-generation drug-eluting stent implantation in patients with diabetes mellitus: A meta-analysis of randomized controlled trials
Source: PLoS One. 2020 Dec 16;15(12):e0242845. doi: 10.1371/journal.pone.0242845 (PMC7743959; doi:10.1371/journal.pone.0242845)

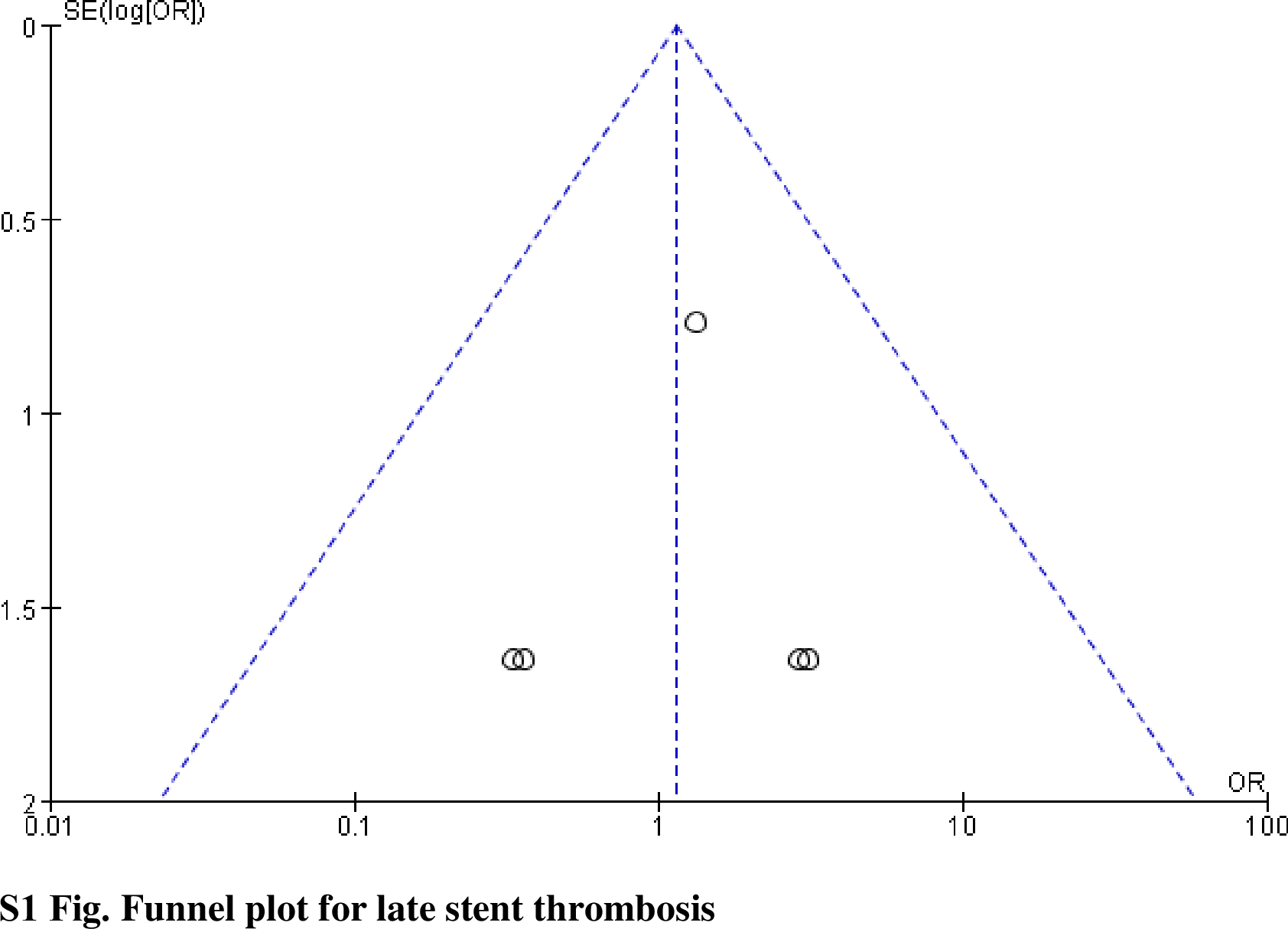

Supplement: S1 Fig — OR: odds ratio; SE: standard error. (TIF) [file pone.0242845.s001.tif]

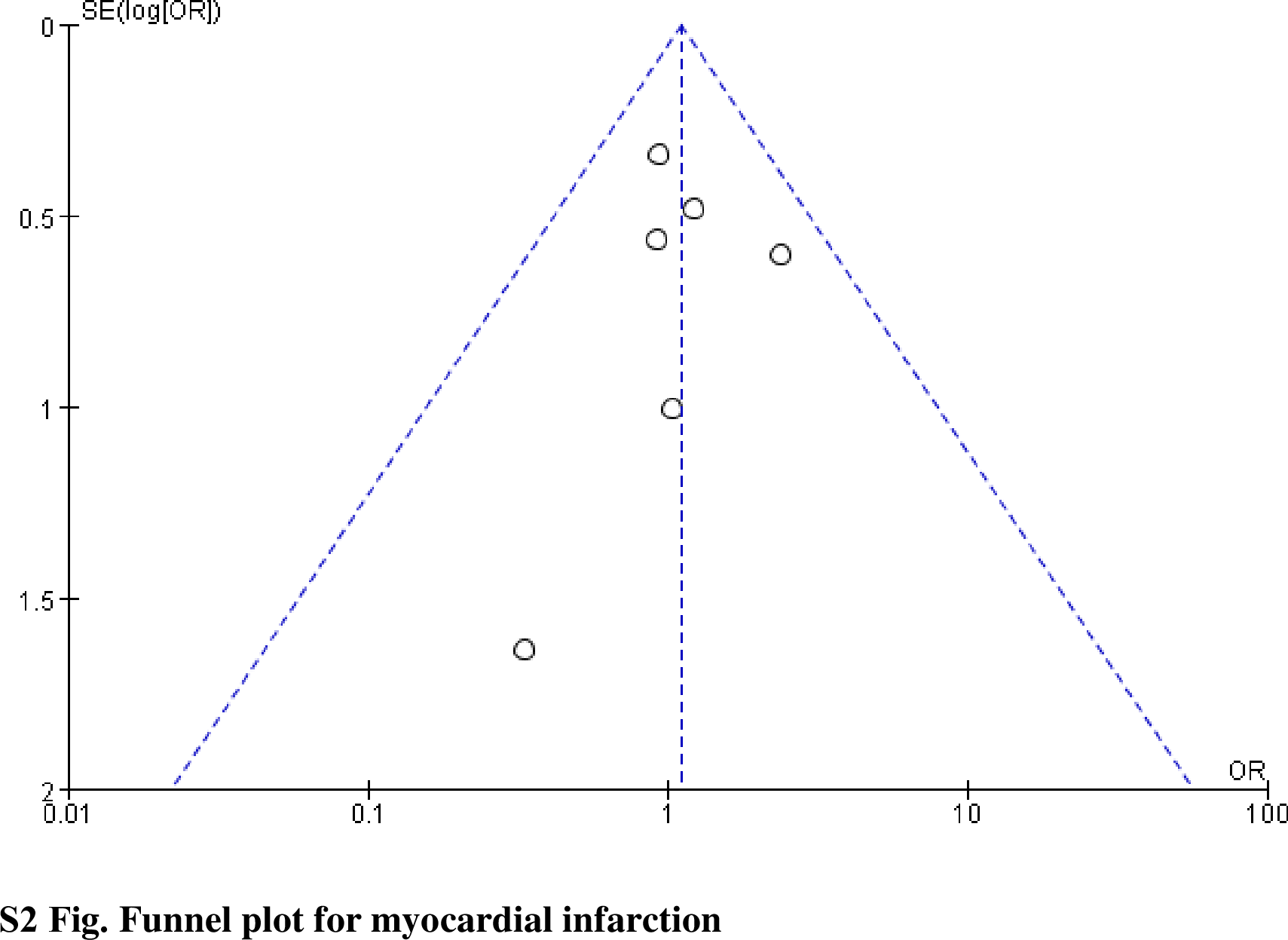

Supplement: S2 Fig — OR: odds ratio; SE: standard error. (TIF) [file pone.0242845.s002.tif]

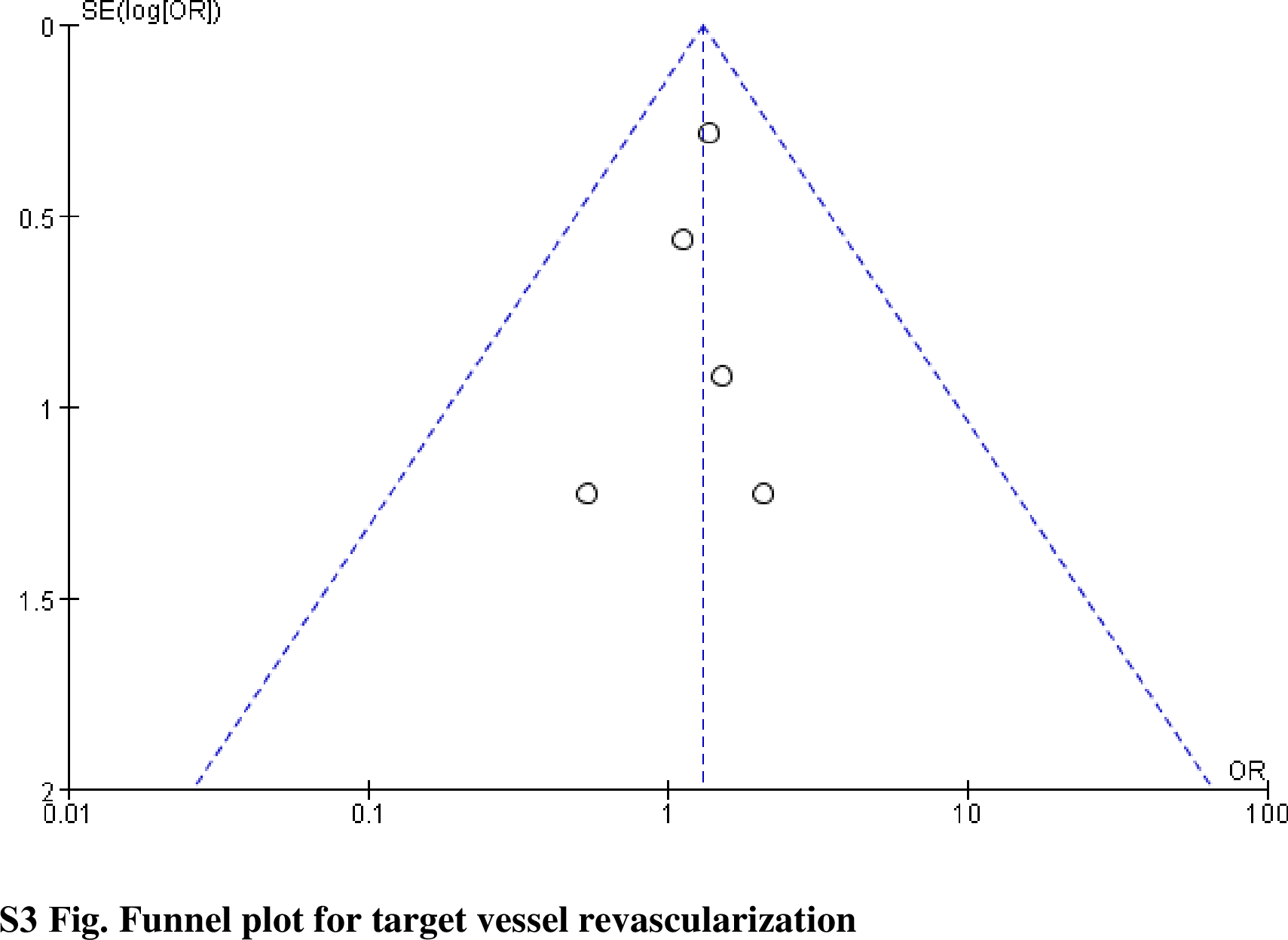

Supplement: S3 Fig — OR: odds ratio; SE: standard error. (TIF) [file pone.0242845.s003.tif]

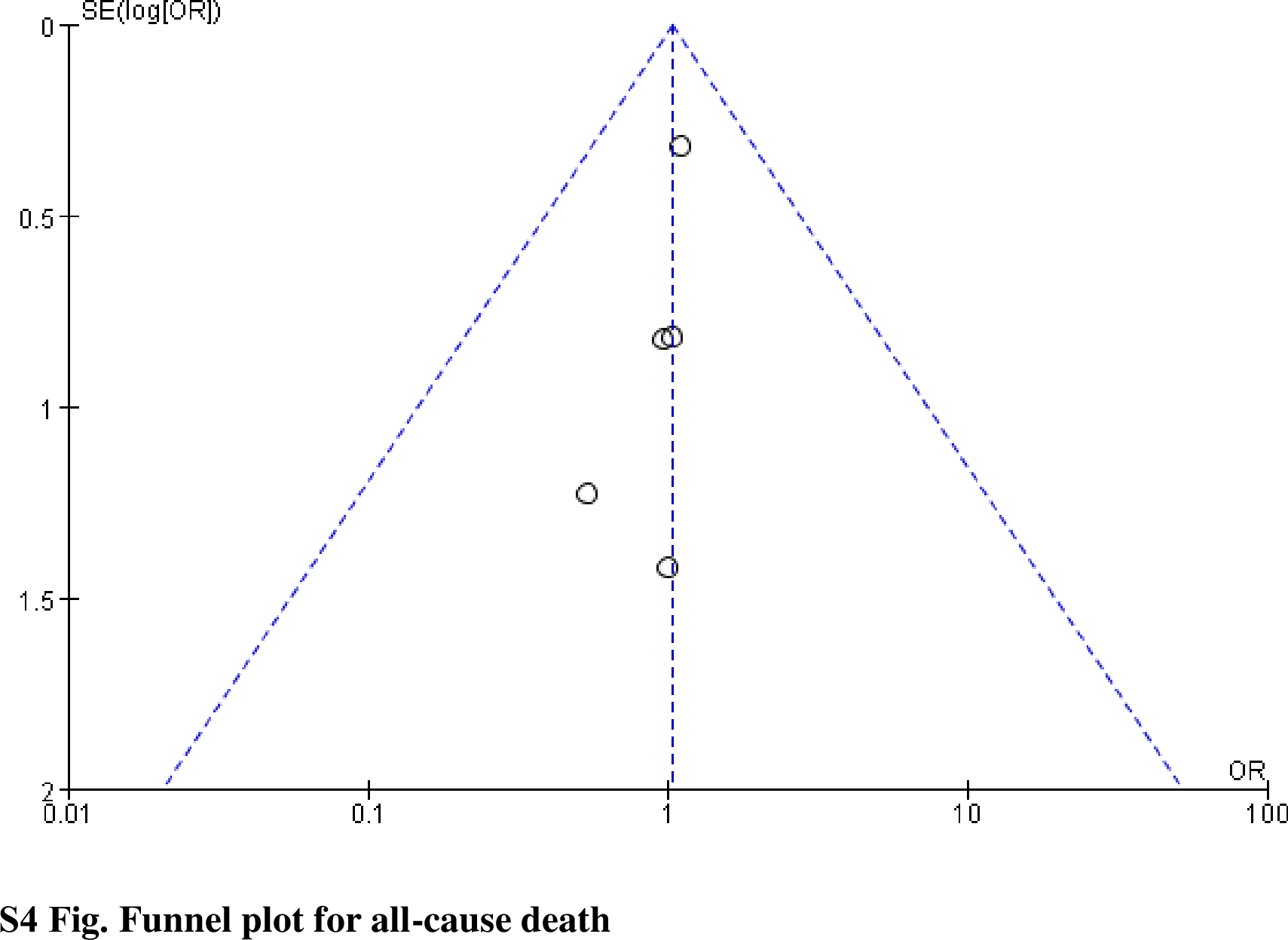

Supplement: S4 Fig — OR: odds ratio; SE: standard error. (TIF) [file pone.0242845.s004.tif]

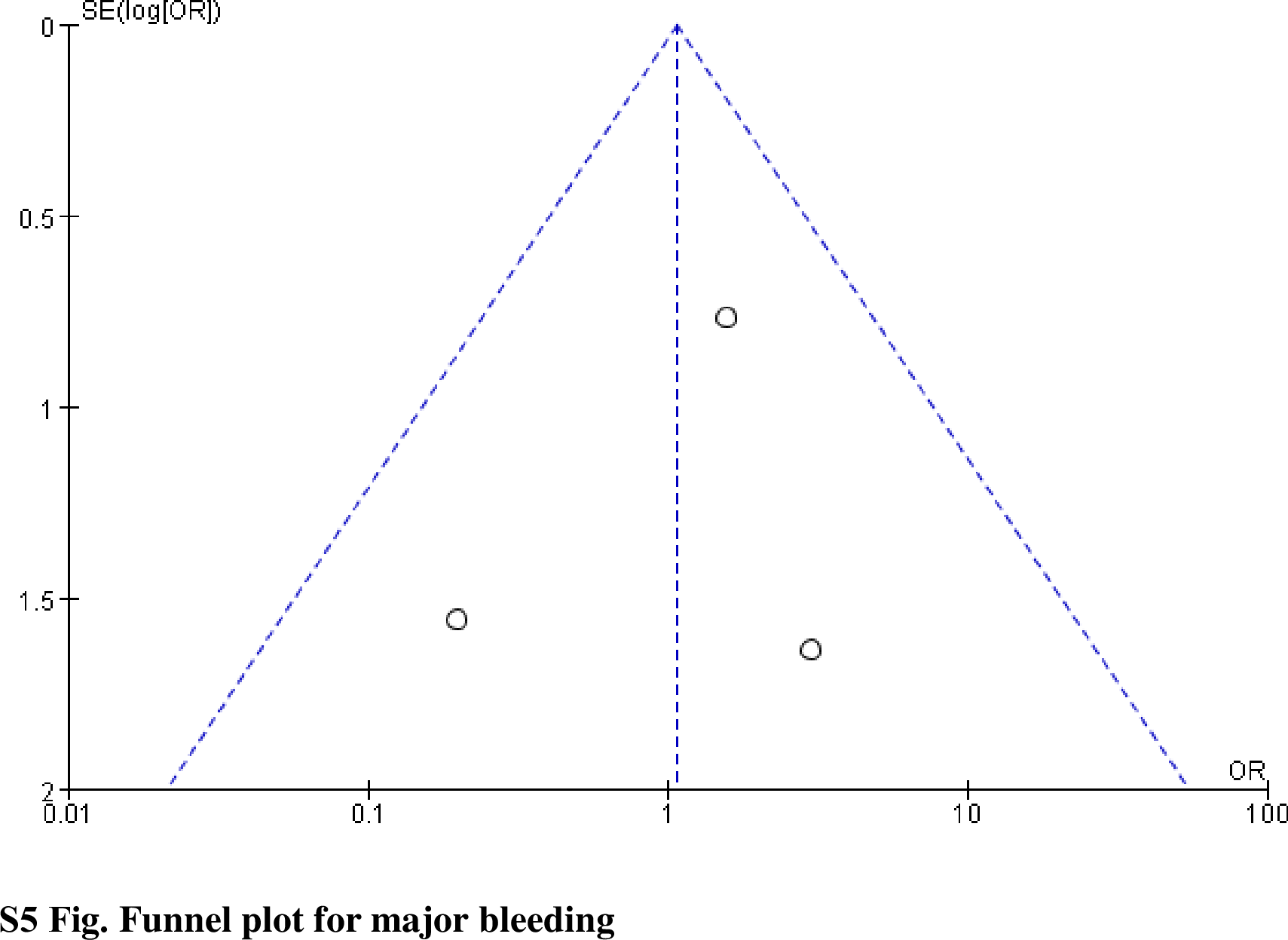

Supplement: S5 Fig — OR: odds ratio; SE: standard error. (TIF) [file pone.0242845.s005.tif]
