# Supplementary material for: Short- versus long-term dual antiplatelet therapy after second-generation drug-eluting stent implantation in patients with diabetes mellitus: A meta-analysis of randomized controlled trials
Source: PLoS One. 2020 Dec 16;15(12):e0242845. doi: 10.1371/journal.pone.0242845 (PMC7743959; doi:10.1371/journal.pone.0242845)
Supplement: S1 Table — (DOCX) [file pone.0242845.s006.docx]

**S1 Table****. Risk of bias assessment in details**

| Study | Random sequence generation | Allocation concealment | Blinding of participants and personnel | Blinding of outcome assessment | Incomplete outcome data | Selective reporting | Other bais |
| --- | --- | --- | --- | --- | --- | --- | --- |
| RESET [10] | low risk: Web-based response system | Yes | High risk: Open-label study | Yes | low risk | low risk | low risk |
| OPTIMIZE [11] | low risk: A dedicated  web-based system | Yes | High risk: Open-label  study | Yes | low risk | low risk | low risk |
| ITALIC [12] | low risk: Web-based system | Yes | High risk: Open-label study | Yes | low risk | low risk | low risk |
| I-LOVE IT 2 [13] | low risk: Web-based allocation system and was stratified by center | Yes | High risk: single-blind | Yes | low risk | low risk | low risk |
| Tarantini2016 [14] | low risk: Web-based case report | Yes | High risk: Open-label  study | Yes | low risk | low risk | low risk |
| SMART-DATE [15] | low risk: Web-based  system by computer-generated block randomisation | Yes | High risk: Open-label study | Yes | low risk | low risk | low risk |
