# Supplementary material for: Short- versus long-term dual antiplatelet therapy after second-generation drug-eluting stent implantation in patients with diabetes mellitus: A meta-analysis of randomized controlled trials
Source: PLoS One. 2020 Dec 16;15(12):e0242845. doi: 10.1371/journal.pone.0242845 (PMC7743959; doi:10.1371/journal.pone.0242845)
Supplement: S1 Text — (DOCX) [file pone.0242845.s008.docx]

**S1 Text. Search strategy**

#1 diabetes mellitus*

#2 diabetes*

#3 #1 OR #2

#4 percutaneous coronary intervention*

#5 drug- eluting stent*

#6 #4 OR #5

#7 dual antiplatelet therapy*

#8 aspirin*

#9 clopidogrel*

#10 prasugrel*

#11 ticagrelor*

#12 P2Y12 receptor inhibitor*

#13 #7 OR #8 OR #9 OR #10 OR #11 OR #12

#14 #3 AND #6 ADN #13
