# Supplementary material for: Short- versus long-term dual antiplatelet therapy after second-generation drug-eluting stent implantation in patients with diabetes mellitus: A meta-analysis of randomized controlled trials
Source: PLoS One. 2020 Dec 16;15(12):e0242845. doi: 10.1371/journal.pone.0242845 (PMC7743959; doi:10.1371/journal.pone.0242845)
Supplement: Attachment — Submitted filename: Response to Reviewers.docx [file pone.0242845.s009.docx]

**Response to Reviewers**

Dear Editor and reviewers:

Thank you for your letter and the reviewers’ comments on our manuscript entitled "Short- versus long-term  dual antiplatelet therapy after second-generation drug-eluting stent implantation in patients with diabetes mellitus: A Meta-analysis of Randomized Controlled Trials". Those comments are very helpful for revising and improving our paper. We have studied the comments carefully and made corrections which we hope meet with approval. The main corrections are in the manuscript and the responds to the editor and reviewers’ comments are as follows.

Reply to editor comments:

1) a subgroup analysis of ACS and SIHD patients

Response: Since the study we included did not report the proportion of diabetic patients with ACS or SIHC, we were unable to conduct a subgroup analysis of such patients. This point has been described in the third point of the limitations of this article

2) 3 months vs 6 months instead of 6 months vs 12 months

Response: Current clinical practice guidelines recommend 6–12-month DAPT following DES implantation. Despite these recommendations, due to refinements in DES technologies and the advent of potent P2Y12 receptor inhibitors, the optimal DAPT after stent implantation in patients with diabetes continues to be a matter of debate. Unfortunately, as literature is scant concerning DAPT durations for DM, the current meta-analysis was hindered in determining the impact of second-generation DESs or new-generation P2Y12 inhibitors on the optimal DAPT duration. We searched a large amount of literatures and found that most trials compared the DAPT of 3-6 months to 12 months or longer. Due to the limited literature available, we could not compare 3 months to 6 months of DAPT. And our meta-analysis shows that DAPT of 3-6 months is not inferior to DAPT of 12-24 months, which also has a certain guiding significance for clinical practice.

Reply to reviewer #1

1. 6 months DAPT after PCI has already been accepted in the guidelines to be safe for all patients including diabetes. The question is not to compare 6 months of DAPT to 12 months or more, but rather to compared shorter DAPT therapy duration to 6 months DAPT therapy duration.

Response: At present, there are few literature about the duration of DAPT after PCI in patients with diabetes, and most trials compared the DAPT of 3-6 months to 12 months or longer, in order to explore the optimal DAPT duration for such patients. Due to limited data, we were unable to compare 6 months with shorter duration of DPAT. And our meta-analysis shows that for patients with stable coronary heart disease or ACS, 3-6 months of DAPT was noninferior to 12-24 months, which also has a certain guiding significance for clinical practice.

2. The manuscript is lacking the brief overview of each research studies used in meta-analysis in tabular format including stent types, IDDM vs NIDDM if data is available, bifurcation stenting, anticoagulation used during PCI, duration of DAPT, follow-up time, etc. This is very vital for any meta-analysis. I see some details in the result section, but that is not enough.

Response: Some tables have been modified, as shown in Table 1 and Table 2.

Reply to reviewer #2

Besides few words corrections, it is a well written nice manuscript. It is written in good intellectual language and lays the strengths and weaknesses of the study. The limitations were laid out nicely and it speaks for the study power.

Response: None.

Once again, thank you very much for your constructive comments and suggestions which would help us both in English and in depth to improve the quality of the paper.

Kind regards,

Corresponding author : junhuang

E-mail address: [junhuang918@163.com](mailto:junhuang918@163.com)

10/30/2020
